# Supplementary material for: Identification and functional analysis of non-coding regulatory small RNA FenSr3 in Bacillus amyloliquefaciens LPB-18
Source: PeerJ. 2023 May 15;11:e15236. doi: 10.7717/peerj.15236 (PMC10194069; doi:10.7717/peerj.15236)
Supplement: Supplemental Information 4 [file peerj-11-15236-s004.zip › KO/CK-vs-T1_map/map04514.html]

KEGG PATHWAY: Cell adhesion molecules (CAMs) - Reference pathway


|  |  |
| --- | --- |
| **Cell adhesion molecules (CAMs) - Reference pathway** |  |

[
Pathway menu
| Organism menu
| Pathway entry
| Show description
| User data mapping
]

Cell adhesion molecules are (glyco)proteins expressed on the cell surface and play a critical role in a wide array of biologic processes that include hemostasis, the immune response, inflammation, embryogenesis, and development of neuronal tissue. There are four main groups: the integrin family, the immunoglobulin superfamily, selectins, and cadherins. Membrane proteins that mediate immune cell–cell interactions fall into different categories, namely those involved in antigen recognition, costimulation and cellular adhesion. Furthermore cell-cell adhesions are important for brain morphology and highly coordinated brain functions such as memory and learning. During early development of the nervous system, neurons elongate their axons towards their targets and establish and maintain synapses through formation of cell-cell adhesions. Cell-cell adhesions also underpin axon-axon contacts and link neurons with supporting schwann cells and oligodendrocytes.

|  |  |  |
| --- | --- | --- |
| Reference pathway | 184% 150% 122% 100% 82% 67% 55% | 图片下载 |
